# Population Dynamics and Cross-Feeding in an Antibiotic-Degrading Synthetic Ecosystem: Insights from Single-Cell Raman Spectroscopy and Stable Isotope Probing

**DOI:** 10.1021/acs.estlett.6c00474

**Published:** 2026-07-27

**Authors:** Rui Xue, Xuan Wu, Rong Ji, Lifeng Lin, Kai Yang, Philippe F.-X. Corvini, Li Cui, Yong-Guan Zhu, Hong-Zhe Li

**Affiliations:** † Chinese Academy of Sciences, Institute of Urban Environment, Xiamen 361021, Fujian, China; ‡ 12581 Nanjing University, School of the Environment, Nanjing 210093, Jiangsu, China; § Hochschule fur Life Sciences FHNW, Muttenz 4132, Basel Landschaft, Switzerland; ∥ Chinese Academy of Sciences, 26442 Research Centre for Eco-Environmental Sciences, Beijing 100085, Beijing, China

**Keywords:** Sulfonamides, Antibiotics, Biodegradation, Bacterial consortium, Commensalism

## Abstract

We report on commensalism enabling the entire degradation of the sulfadiazine antibiotic by a consortium composed of *Microbacterium* sp. strain BR1 and *Terrabacter*. ^13^C,^15^N-labeled sulfadiazine was synthesized and used as sole source of carbon in axenic cultures of *Microbacterium* and *Terrabacter* as well as cocultures of both genera. HPLC analyses showed that *Microbacterium* degraded sulfadiazine with accumulation of 2-aminopyrimidine, while the *Terrabacter* culture did not degrade sulfadiazine. The consortium degraded labeled sulfadiazine without any accumulation of 2-aminopyrimidine. Cross-feeding was efficiently synchronized in the synthetic consortium, and the release of 2-aminopyrimidine from sulfadiazine by *Microbacterium* is a prerequisite for its degradation by *Terrabacter*. Raman spectra in the fingerprint region enabled the discrimination of the two genera at the single cell level and the calculation of the cell number ratio, providing quantitative insight into the population dynamics in coculture. The cell counts in axenic culture and coculture were consistent with HPLC analyses, reflecting their metabolic activities and trophic interactions. Stable isotope probing and subsequent analyses of Raman spectra of *Terrabacter* in the synthetic consortium revealed a significant shift from 1000 cm^–1^ to 988 cm^–1^, proving that this bacterium assimilates 2-AP for biomass synthesis and that less than 50% of *Terrabacter* cells were degrading 2-AP.

## Graphical abstract

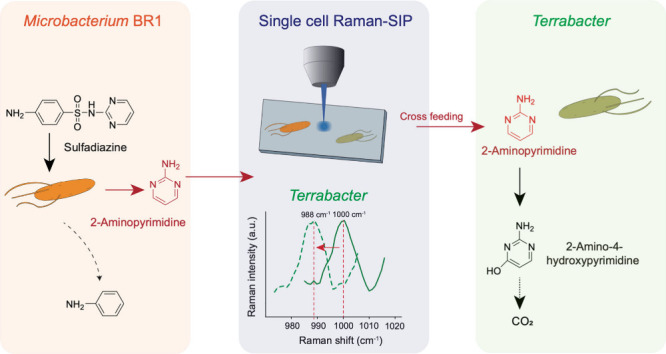

## Introduction

Cross-feeding plays an important role in shaping and stabilizing microbial communities.^1^ Microorganisms can engage in commensal interactions to remove toxic metabolites from the environment.^2^ Although antibiotic catabolism may reduce the selective pressure imposed by residual antibiotics, it may also indirectly affect ARG dissemination by enriching antibiotic-degrading populations that carry ARGs or mobile genetic elements. While the spreading of antibiotic resistance in the environment is intensively monitored,^3^ growing awareness about the catabolism of antibiotics by microorganisms adds to the complexity of understanding the interaction between this detoxification mechanism and the spreading of antibiotic resistance genes.^4,5^

Cross-feeding is necessary to remove the various moieties of sulfonamide complex molecules like sulfamethoxazole in activated sludge microbiome.^6^ A decade ago, *Microbacterium lacus* was isolated from a soil mesocosm for its capacity to assimilate the aniline moiety of sulfadiazine leaving 2-aminopyrimidine (2-AP) the heteroatom-bearing moiety as dead-end metabolite.^7^ 2-AP results from *ipso*-substitution degradation reaction first described in *Microbacterium* BR1.^8^ Further study led to the isolation of *Terrabacter*-like bacterium that is capable to mineralize 2-AP from the same mesocosm and led to the assumption that cross-feeding with *Microbacterium lacus* would occur.^9^ Although cross-feeding during sulfadiazine (SDZ) and, more generally, sulfonamide degradation has been recognized, it remains unclear how this process shapes cell-to-cell interactions within bacterial communities at the single-cell level. Specifically, quantitative and isotope-level evidence is still needed to identify the active populations involved in antibiotic-derived substrate assimilation and to determine how their contributions change during coordinated SDZ degradation.

Here, we describe how fingerprint single-cell Raman spectroscopy and stable isotope probing were useful to decipher cross-feeding and monitor population dynamics in an antibiotic-degrading microbial community consisting of *Microbacterium* BR1 and *Terrabacter*. We synthesized ^13^C–^15^N-labeled sulfadiazine starting from [^13^C,^15^N_3_]-guanidine hydrobromide for stable isotope probing. We prepared triplicates of coculture and single cultures of those microorganisms in medium containing pyrimidine ^13^C–^15^N-labeled sulfadiazine (1 mM) as sole source of carbon. Samples were taken at days zero, two, and four for HPLC analyses to determine the remaining concentrations of SDZ and 2-AP in culture supernatants. Cell suspension samples were analyzed using a flow-cytometer and the Raman microspectroscopy described previously^10^ to distinguish each genus and assess at single cell level their growth in culture as well as their relative abundance in coculture. The analysis of stable-isotope-labeled single-cell Raman spectroscopy revealed a ^15^N and ^13^C-induced spectral shift resulting from the assimilation of ^15^N and ^13^C atoms of 2-AP into the biomass of *Terrabacter*, demonstrating their *in situ* degradation activity within the artificial consortium.

## Materials and Methods

### Synthesis of ^13^C,^15^N-Labeled Sulfadiazine

#### Design Rationale and Synthesis of Pyrimidine-Ring-Labeled Sulfadiazine

^13^C,^15^N-labeled sulfadiazine was synthesized to enable single-cell tracing of sulfadiazine-derived cross-feeding between *Microbacterium* BR1 and *Terrabacter.* The isotope labels were specifically introduced into the pyrimidine-derived 2-aminopyrimidine moiety because this fragment is released from sulfadiazine by *Microbacterium* BR1 and subsequently utilized by *Terrabacter*. Therefore, pyrimidine-ring labeling allowed us to trace the transfer and assimilation of the cross-fed 2-aminopyrimidine intermediate by *Terrabacter*. In contrast, labeling the aniline/sulfonamide moiety would mainly report the metabolism of *Microbacterium* BR1, while uniformly labeled sulfadiazine would obscure the strain-specific interpretation of isotope incorporation.

Although dual ^13^C/^15^N labeling complicates the assignment of individual Raman band shifts to either ^13^C or ^15^N incorporation, this design increases the isotope-induced spectral contrast for detecting the assimilation of the labeled 2-aminopyrimidine-derived atoms into cellular biomass. Thus, the dual-labeling strategy was used to identify active cells involved in cross-feeding rather than to separately quantify carbon and nitrogen assimilation. Raman spectral shifts detected in *Terrabacter* cells therefore indicate the single-cell assimilation of sulfadiazine-derived 2-aminopyrimidines.

The labeled sulfadiazine was synthesized through a three-step route using [^13^C,^15^N_3_]-labeled guanidine hydrobromide as the precursor. Briefly, [^13^C,^15^N_3_]-labeled 2-aminopyrimidine was first prepared and subsequently coupled with N-acetylsulfanilyl chloride, followed by alkaline hydrolysis and recrystallization to obtain [^13^C,^15^N_3_]-labeled sulfadiazine. The final product had a purity of 97.0%, as determined by HPLC, with an overall yield of 61.2% from [^13^C,^15^N_3_]-labeled 2-aminopyrimidine. Full synthetic and analytical details are provided in the Supporting Information.

### Cultures of *Microbacterium* and *Terrabacter*

Preculture of *Microbacterium* sp. BR1 isolated in a previous study^11^ was prepared using 20% glycerol stock to inoculate supplemented minimal salt medium (MSM Brunner) supplemented with 25% Luria–Bertani (LB) and 1 mM sulfadiazine. Preculture of *Terrabacter* (DSMZ 28514) was prepared using glycerol stock to inoculate supplemented MSM Brunner supplemented with 25% LB. After 28 h of incubation at 28 °C on an orbital shaker, precultures of each bacterium were used to inoculate MSM Brunner supplemented with vitamins^8^ and 1 mM ^13^C- and ^15^N-diazine labeled sulfadiazine was inoculated with *Microbacterium* OD_600 nm_ 0.05 and *Terrabacter* OD_600 nm_ 0.05. To adjust the initial OD_600 nm_ to 0.05, the OD_600 nm_ of each preculture was measured and corresponding volume of each preculture was taken. The cells were centrifuged and rinsed twice with 0.85% NaCl water prior to inoculation of the coculture. The coculture was incubated at 28 °C on an orbital shaker. Various controls were prepared. A coculture containing nonlabeled sulfadiazine was prepared. Axenic cultures of *Microbacterium* and *Terrabacter* incubated with 1 mM ^13^C-diazine labeled sulfadiazine were also prepared. All the cultures were prepared in triplicates. Samples were taken at day 0, 2, and 4 to measure (i) the concentration of SDZ and 2-aminopyridine using HPLC; (ii) determine the ratio of *Microbacterium* and *Terrabacter* cells using Raman microscopy in the fingerprint region; (iii) perform cell counts using flow cytometry and (iv) analyze chemical shifts using Raman microscopy.

### HPLC Analyses

HPLC analyses were performed as described by Tappe et al. (2015) using Hitachi L2000 HPLC system and Daisopak column (Japan, Caoda Corporation LTD). The UV detector was set at 267 nm for SDZ and at 298 nm for 2-AP.

### Raman Confocal Microscopic Analyses

Bacterial samples were prepared by washing the bacterial suspension three times with sterile water to remove residual culture media. A 2 μL aliquot of the bacterial suspension was spotted onto aluminum foil and allowed to air-dry at room temperature prior to Raman analysis. Single-cell Raman spectra were collected using a LabRAM Aramis confocal Raman microscope (HORIBA Jobin-Yvon) equipped with a 532 nm Nd:YAG laser, 300 g/mm grating, and a 100× air objective (Olympus, NA = 0.09). Spectra were acquired over the range of 500–3200 cm^–1^ with an acquisition time of 9 s, collecting approximately 100 spectra per sample. All spectra were subsequently processed using LabSpec5 software (HORIBA Jobin-Yvon) for baseline correction and normalization. Individual bacterial cells were assigned to *Terrabacter* or *Microbacterium* based on the presence and relative intensity of the diagnostic Raman fingerprint features identified from the axenic cultures. Raman-shift-positive cells were classified according to predefined diagnostic peak-position criteria. Diagnostic bands were selected based on well-established microbial Raman peak assignments reported in previous studies.^12^

### Cell Count Measurement Using Flow Cytometry

Cell abundance was quantified by flow cytometry based on forward- and side-scatter signals. Sterile medium controls were analyzed under the same acquisition settings to define background signals and instrument noise. Bacterial events were gated using FSC/SSC plots, with the gate set above the background region observed in sterile medium controls. Events within the background/noise region were excluded. Doublets and aggregates were further excluded using FSC-A/FSC-H and SSC-A/SSC-H gating. The same acquisition settings, flow rate, acquisition volume, and gating template were applied to all samples. Cell concentrations were calculated from the number of gated bacterial events per acquired volume and expressed as cells μL^–1^.

## Results and Discussion

In previous studies, the degradation capacity and metabolic products of *Microbacterium* BR1 and *Terrabacter* for sulfadiazine (SDZ) have been thoroughly reported.^8,9^ *Microbacterium* is capable of degrading SDZ and accumulating 2-amino-5-pyrimidine (2-AP) as a dead-end metabolite, while *Terrabacter* further hydroxylates, cleaves the aromatic ring, and mineralizes 2-AP. The synergistic coculture of these two strains facilitates the complete degradation of SDZ, as shown in Figure 1. HPLC-DAD analyses showed that only cocultures of *Microbacterium* BR1 and *Terrabacter* synergistically degraded both 4-AP (released aniline moiety of SDZ) and 2-AP within 4 days (Table 1).

**Figure 1:**
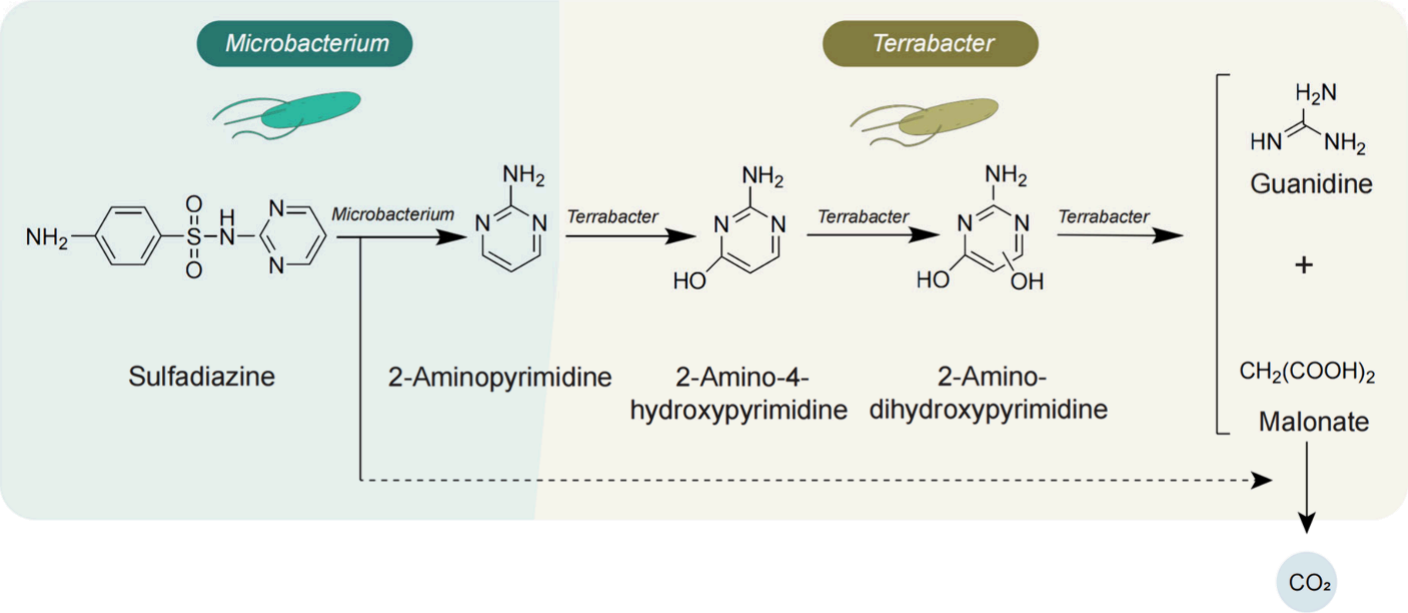
Sequential degradation of sulfadiazine (SDZ) by *Microbacterium* and *Terrabacter* bacteria. *Microbacterium* (left) cleaves SDZ into 2-aminopyrimidine (2-AP). *Terrabacter* (right) then degrades 2-AP through hydroxylation to 2-amino-4-hydroxypyrimidine, followed by further hydroxylation and pyrimidine ring cleavage, resulting in the formation of guanidine and malonate, which are further mineralized to CO_2_.

**Table 1:** Concentration of Sulfadiazine (SDZ) and 2-Aminopyrimidine (2-AP) in Culture Supernatants^a^

	*Microbacterium* + *Terrabacter*		*Microbacterium*		*Terrabacter*	
Days	SDZ (mM)	2-AP (mM)	SDZ (mM)	2-AP (mM)	SDZ (mM)	2-AP (mM)
0	1.0 ± 0.1	0.0	1.0 ± 0.1	0.0	1.0 ± 0.1	0.0
2	0.59 ± 0.18	0.0	0.0	1.0 ± 0.1	1.0 ± 0.1	0.0
4	0.0	0.0	0.0	1.0 ± 0.1	1.0 ± 0.1	0.0

a Values are presented as mean ± SD from three independent biological replicates.

HPLC-based time-course analyses showed distinct SDZ degradation patterns among the axenic and coculture systems (Table 1). Axenic cultures of *Microbacterium* led to the entire degradation of sulfadiazine with concomitant accumulation of 2-AP within 2 days, while axenic cultures of *Terrabacter* left SDZ unaltered after 4 days. This showed that *ipso*-substitution reaction carried out by *Microbacterium* is a prerequisite to the biodegradation of 2-AP by *Terrabacter*. In the cocultures, sulfadiazine concentration was still 0.59 ± 0.18 mM after 2 days. Although, the sulfadiazine biodegradation kinetics was faster in *Microbacterium* cultures, there was accumulation of 2-AP as dead-end product. In the coculture, the slightly slower SDZ removal was likely related to the lower initial abundance of *Microbacterium*. No traces of 2-AP were detected in cocultures at all sampling times indicating a synchronized cross-feeding. The stable assembly of this synthetic consortium enabled the degradation of 2-AP, a SDZ transformation product which deserve toxicological studies, and is of relevance for agricultural practices involving the application of sulfadiazine-containing manure as soil fertilizer.

The in-depth analyses of the fingerprints in Raman spectra of single cells from the axenic cultures allowed for discrimination of both strains in coculture based on their characteristic chemical compositions. These two strains exhibited distinct Raman fingerprint peaks at 1165 cm^–1^ and 1534 cm^–1^ (Figure 2a), which are consistent with carotenoid-associated Raman features mainly reflecting C–C and C=C stretching vibrations of the conjugated polyene backbone, respectively.

**Figure 2:**
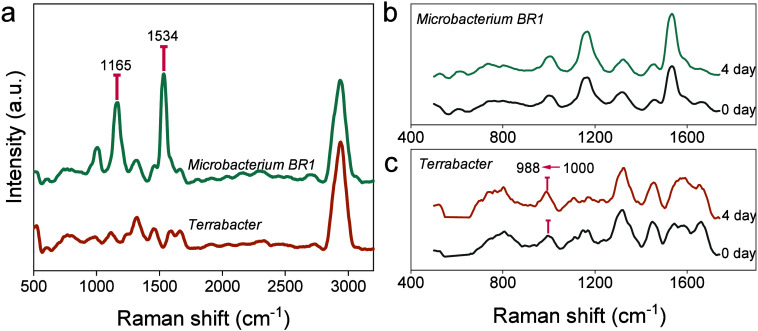
(a) Raman spectra of *Microbacterium* BR1 and *Terrabacter*. Average single-cell Raman spectra of *Microbacterium* BR1 (b) and *Terrabacter* (c) after 4 days of incubation in a coculture system containing ^13^C-labeled sulfadiazine. All spectra represent the averages of 20 single-cell measurements.

The cell counts of *Microbacterium* BR1 and *Terrabacter* in axenic and cocultures were carried out using flow cytometry (Figure 3a). In axenic cultures of *Microbacterium* BR1, the cell count decreased from 1067 ± 206 (day 0) to 832 ± 89 cells/μL (day 2), while *Terrabacter* culture decreased from 775 ± 10 (day 0) to 474 ± 44 cells/μL (day 2).

**Figure 3:**
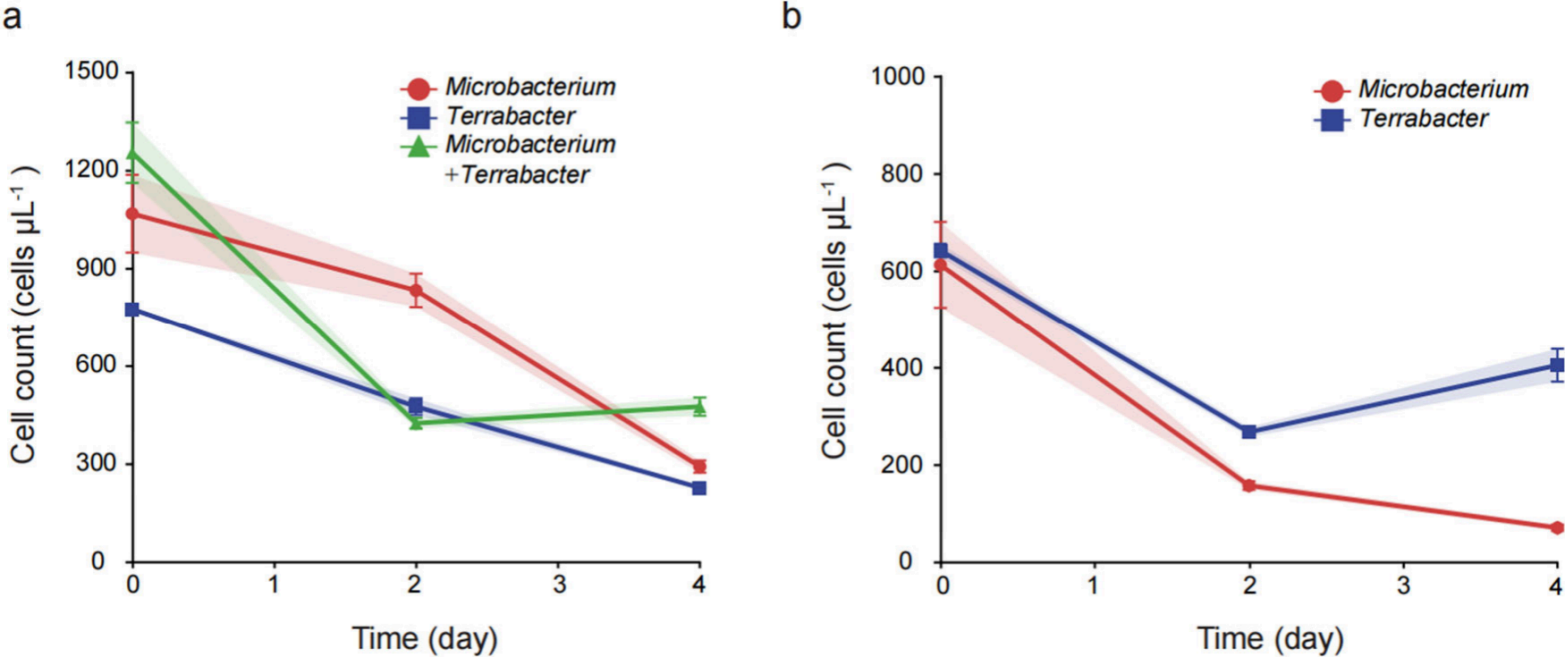
Evolution of cell counts of *Microbacterium* and *Terrabacter* in axenic and coculture and individual population in the coculture. (a) Total cell counts of axenic cultures and coculture, which were determined using flow cytometry. (b) Cell counts of *Microbacterium* and *Terrabacter* in the synthetic consortium. The cell counts of each genus were calculated by multiplying the total cell count of the coculture by the relative proportions of *Microbacterium* and *Terrabacter*, which were determined using Raman microscopy in the fingerprint region.

The faster decrease in the population of *Terrabacter* than that of *Microbacterium* can be explained by the fact that *Terrabacter* alone was not able to feed on SDZ molecule, while *Microbacterium* BR1 subsisted on 4-AP moiety of SDZ. The initial decline in *Terrabacter* abundance may therefore reflect the combined effects of SDZ-associated antibiotic stress and nutrient limitation. In the coculture, the total cell count decrease was less pronounced as it evolved from 1253 ± 160 cells/μL (day 0) to 423 ± 28 (day 2) to eventually 541 ± 9 cells/μL (day 4) (Figure 3b). The subsequent increase in cell count is consistent with the assimilation of *Microbacterium*-derived transformation products by *Terrabacter*, which may have supported its recovery and growth in the later stage of incubation. Moreover, from day 2 to day 4, *Microbacterium* abundance declined more slowly in the coculture than in the axenic *Microbacterium* culture, suggesting a potential protective effect of *Terrabacter* through the removal of 2-AP. It was possible to evaluate the relative population of *Microbacterium* BR1 and *Terrabacter* cells through the analysis of microscope images acquired using the Raman microspectroscopy. During the coculture of *Microbacterium* and *Terrabacter*, the proportion of *Terrabacter* cells increased over the course of the degradation process, starting at 51.7 ± 5.8% on Day 0, rising to 63.0 ± 2.0% on Day 2, and reaching 85.0 ± 3.6% on Day 4. These results indicated that the slight regrowth in the coculture was due to the growth of *Terrabacter* from 267 ± 18 (Day 2) to 404 ± 59 cells/μL (Day 4), while the population of *Microbacterium* decreased from 157 ± 15 (Day 2) to 70 ± 11 (Day 4). These data show clear population dynamics with *Terrabacter* becoming increasingly dominant over time, which fits the cross-feeding relationship, where *Terrabacter* is dependent on the preliminary release of 2-AP by *Microbacterium* BR1. Based on the stable isotope probing approach, no Raman shift was observed in the axenic cultures. While discriminating *Terrabacter* from *Microbacterium* BR1 cells using the characteristic spectral feature, the analysis of cocultures showed a shift in the spectra of *Terrabacter* cells from 1000 to 988 cm^–1^ (Figure 2c). This shift was observed on Day 2 and Day 4, where 42.8 ± 1.8% and 45.9 ± 3.2%, respectively, of the total number of cells of *Terrabacter* showed a band shift in their Raman spectra, meaning that less than half of this genus was involved in the assimilation of 2-AP. This partial response may reflect single-cell physiological heterogeneity within the *Terrabacter* population. Actively growing or dividing cells may have a greater capacity to take up and metabolize 2-AP, resulting in a detectable Raman shift, whereas cells in stationary or low-metabolic states may not exhibit sufficiently strong spectral changes. Therefore, the ∼45% Raman-positive fraction suggests that 2-AP degradation in *Terrabacter* may be primarily driven by a metabolically responsive subpopulation rather than by uniform activation of the entire population. Such metabolic heterogeneity may enhance the stability of the consortium by allowing a responsive subpopulation to drive 2-AP degradation while maintaining a low-activity fraction that could buffer fluctuations in substrate availability or environmental stress. In addition, cells with low levels of isotope incorporation may also have contributed to 2-AP transformation, but their assimilation signals may have remained below the detection threshold of Raman SIP. No isotope-induced Raman band shift was detected for *Microbacterium* BR1 (Figure 2b). In contrast, a clear shift of the Raman band near 1000 cm^–1^ was observed for *Terrabacter*. This band is commonly assigned to the phenylalanine ring-breathing mode and mainly reflects vibrations of the aromatic carbon skeleton, indicating that the observed isotopic shift is attributable to ^13^C incorporation into cellular biomass rather than from ^15^N assimilation.^12^ Therefore, the observed shift provides evidence that *Terrabacter* assimilated carbon derived from ^13^C,^15^N-labeled 2-AP during biomass growth. Combined with the HPLC analyses showing 2-AP degradation, these results support the assimilation of 2-AP-derived atoms by *Terrabacter*. Given the double labeling of the substrate with ^13^C and ^15^N, and the predominant sensitivity of the 1000 cm^–1^ band to ^13^C rather than ^15^N incorporation, we did not assign this specific Raman shift to ^15^N incorporation. Instead, isotope labeling was used as a sensitive approach to visualize substrate-derived biomass assimilation at the single-cell level.

This study investigated cross-feeding of a sulfonamide antibiotic in a synthetic bacterial community. It assessed trophic interactions between the members of this synthetic bacterial community in terms of carbon and nitrogen assimilation, cell growth, and antibiotic degradation kinetics. Future work might consider the assessment of cross-feeding in microbial communities with higher biodiversity to understand how micropollutants can be degraded.

## Supplementary Material



## Notes

Supplementary experimental procedures: synthesis of ^13^C,^15^N-labeled sulfadiazine and validation of synthesized isotope-labeled sulfadiazine by LC-MS, synthetic pathway of [^13^C,^15^N_3_]-labeled sulfadiazine, LC-MS analysis of unlabeled sulfadiazine, LC-MS spectrum of unlabeled sulfadiazine, LC-MS analysis of isotope-labeled sulfadiazine, LC-MS verification of isotope labeling position in sulfadiazine, Raman spectra of *Microbacterium* BR1 and *Terrabacter*, materials, and methods, including site description, sampling of soil and residue, ^15^N tracing experiment, soil and residue chemical analyses, soil microbial analyses, data calculation and statistical analyses, data on biochemical properties of soils, results of two-way ANOVA testing the effects of sampling site, residue carbon level, and their interaction on RE_GNM_, RE_N2_O, and RE_N2_O-related gene abundances, visualizations of N_2_O emissions, and changes in RE_GNM_ and RE_N2_O-related gene abundances in two soils following residue carbon addition (PDF)
