# Supplementary material for: Population Dynamics and Cross-Feeding in an Antibiotic-Degrading Synthetic Ecosystem: Insights from Single-Cell Raman Spectroscopy and Stable Isotope Probing
Source: Environ Sci Technol Lett. 2026 Jul 27;13(8):1122–6. doi: 10.1021/acs.estlett.6c00474 (PMC13472149; doi:10.1021/acs.estlett.6c00474)
Supplement: Supplementary file 1 [file ez6c00474_si_001.pdf]

Supporting information

Population Dynamics and Cross-Feeding in an Antibiotic-Degrading Synthetic Ecosystem: Insights from Single-Cell Raman Spectroscopy and Stable Isotope Probing

[1,4]Xue R, [2]Wu X, [2]Ji R, [1]Lin LF, [1]Yang K, [2,3*]Corvini PF-X, [1]Cui L, [1]Zhu YG, [1]Li HZ

1 Chinese Academy of Sciences, Beijing 100864, Beijing, China;

2 Nanjing University, Nanjing 210093, Jiangsu, China;

3 Hochschule fur Life Sciences FHNW, Muttenz 4132, Basel Landschaft, Switzerland;

4 University of Chinese Academy of Sciences, Beijing 100049, Beijing, China

** Corresponding author: philippe.corvini@fhnw.ch, +41 61 228 54 85*

Experimental procedures

Synthesis of ^{13}C-^{15}N-labelled sulfadiazine

Synthesis of ^{13}C-^{15}N-labelled sulfadiazine was carried out in three steps using [^{13}C, ^{15}N]-labelled guanidine as a precursor, according to the procedure shown in Fig. SI-1.

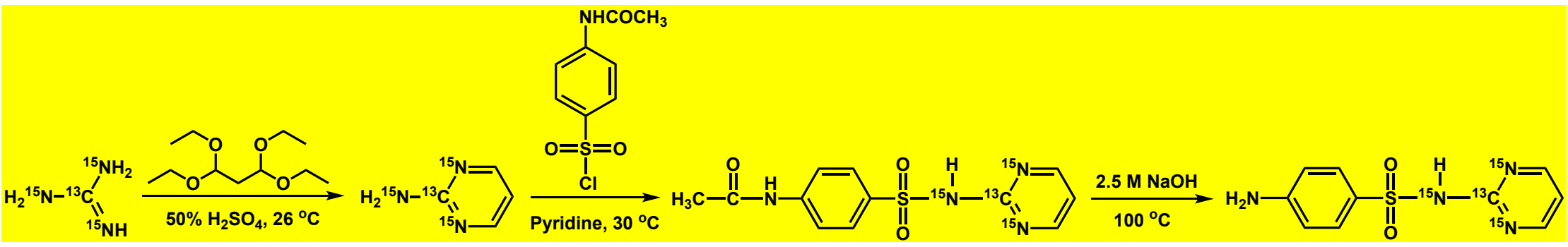

Figure SI-1. Synthetic pathway of [^{13}C,$^{15}N_3$]-labeled sulfadiazine

[^{13}C,$^{15}N_3$]-Labeled 2-aminopyrimidine

[^{13}C,$^{15}N_3$]-Labeled guanidine hydrobromide (850 mg, 5.90 mmol, >99.9% purity, 98.3% of ^{13}C atom, and 98.3% of ^{15}N atom; ALSACHIM, France), and 1,1,3,3-tetraethoxypropane (1.70 g, 7.72 mmol) were added sequentially to sulfuric acid solution (50%, g/g, 35 mL) with stirring in an ice bath (0 °C). The mixture was stirred for 30 h at 26 °C, and then extracted three times with ethyl ether (30 mL each) to remove the excess of 1,1,3,3-tetraethoxypropane. The extracted sulfuric acid solution containing the product was alkalized to pH 13–14 with a NaOH solution (4 M) in an ice bath, and further extracted eight times with ethyl acetate (40 mL each). The extracts were dried with anhydrous Na_2SO_4 and evaporated to ~2 mL. The crude product in the concentrated extract was purified on a pre-packed silica gel column (40 g, 40–60 µm; Agela, Tianjin, China) using a medium pressure preparative chromatography system (CHEETAH TMMP100; Agela, Tianjin, China) equipped with a UV detector and a fraction collector. The UV detection was set at 230 nm and 254 nm. The eluent ran at 15 mL/min, consisting of *n*-hexane (A) and ethyl acetate (B). The elution gradient started with 0% B for 10 min, directly increased to 20% B, kept at 20% B for 15 min, linearly increased to 30% B within 10 min, kept at 30% B for 10 min, linearly increased to 40% B within 10 min, kept at 40% B

for 30 min, linearly increased to 50% B within 10 min, kept at 50% B for 20 min, linearly increased to 80% B within 10 min, and kept at 80% B for 60 min. The eluent between 110–150 min was collected and evaporated under vacuum to dryness. The product [^{13}C,$^{15}N_3$]-labeled 2-aminopyrimidine (329 mg, 3.32 mmol) was obtained in 56.3% yield, with a purity of 98% as determined by high-performance liquid chromatography (HPLC) (See below).

[^{13}C,$^{15}N_3$]-Labeled N-*acetylsulfadiazine*

[^{13}C,$^{15}N_3$]-Labeled 2-aminopyrimidine (296 mg, 2.99 mmol) and *N*-acetylsulfanilyl chloride (1048 mg, 4.48 mmol) were added sequentially to anhydrous pyridine (3.6 mL) with stirring in an ice bath. The mixture was then stirred for 24 h at 30 °C in a water bath. After evaporation of the pyridine, crystallization in ice-cold water, filtration and washing with ice-cold water three times (5 mL each), the wet product [^{13}C,$^{15}N_3$]-labeled *N*-acetylsulfadiazine was obtained with a purity of 95% as determined by HPLC (See below).

[^{13}C,$^{15}N_3$]-Labeled N-*sulfadiazine*

The wet product [^{13}C,$^{15}N_3$]-labeled *N*-acetylsulfadiazine without further purification was hydrolyzed in NaOH solution (2.5 M, 3 mL) for 3 h at 100 °C. After cooling to room temperature, the mixture was neutralized to pH 6 with 6 M HCl and cooled in an ice bath. The precipitates were filtered, washed with ice-cold water three times (5 mL each) to obtain the crude product [^{13}C,$^{15}N_3$]-labeled sulfadiazine. The crude product was recrystallized from boiling methanol. After cooling in an ice bath, the precipitate was centrifuged and washed three times with methanol, resulting in [^{13}C,$^{15}N_3$]-labeled sulfadiazine (465 mg, 1.83 mmol), with a purity of 97.0% as determined by HPLC (See below). The overall yield of [^{13}C,$^{15}N_3$]-labeled sulfadiazine from [^{13}C,$^{15}N_3$]-Labeled 2-aminopyrimidine (the last two synthetic steps) was 61.2%.

High-performance liquid chromatography (HPLC)

The purity of the products was determined using an Agilent HPLC 1100 system (Agilent Technology, USA). Chromatographic separation was achieved by injecting the sample (10 µL) onto an Eclipse XDB-C18 column (250 mm × 4.6 mm, 5 µm; Agilent) at 30 °C with a guard column (4.6 mm × 12.5 mm, 5 µm; Agilent). The flow-rate was set at 1 mL/min. The mobile phase for the analysis of [^{13}C,$^{15}N_3$]-labeled 2-aminopyrimidine consisted of H_2O (80%) and CH_3OH (20%) running isocratically. The mobile phase used to determine the purity of [^{13}C,$^{15}N_3$]-labeled *N*-acetylsulfadiazine and sulfadiazine consisted of H_2O (60%) and CH_3CN (40%) running isocratically. Purity was calculated according to the area normalization method at 230 nm and 270 nm.

Validation of synthesized isotope-labeled sulfadiazine by LC-MS

To verify the molecular mass and fragmentation pattern of the synthesized isotope-labeled sulfadiazine, the compound was analyzed by LC- MS using a Thermo Scientific Orbitrap Exploris 240 mass spectrometer equipped with a heated electrospray ionization source (H-ESI). Chromatographic separation was achieved on a Shimadzu Shim-pack GIST-HP C18 column (2.1 × 100 mm, 3 µm).

MS analysis was conducted in positive ionization mode (Figures SI 2-5). The main source parameters were as follows: spray voltage, 3400 V; auxiliary gas, 2 arbitrary units; sweep gas, 0 arbitrary units; ion transfer tube temperature, 320 °C; and vaporizer temperature, 0 °C. Full-scan ddMS2 data were collected over an m/z range of 100-1000 with an Orbitrap resolution of 60000. Tandem MS spectra were obtained using a data-dependent ddMS^2 method, with a maximum of 20 dependent scans per acquisition cycle. Fragment ions were generated by HCD using stepped collision energies of 10, 20, and 30%, and MS spectra were recorded at an Orbitrap resolution of 60000. The observed accurate precursor ion and diagnostic fragment ions were used to confirm the expected isotope incorporation and labeling position.

Figure

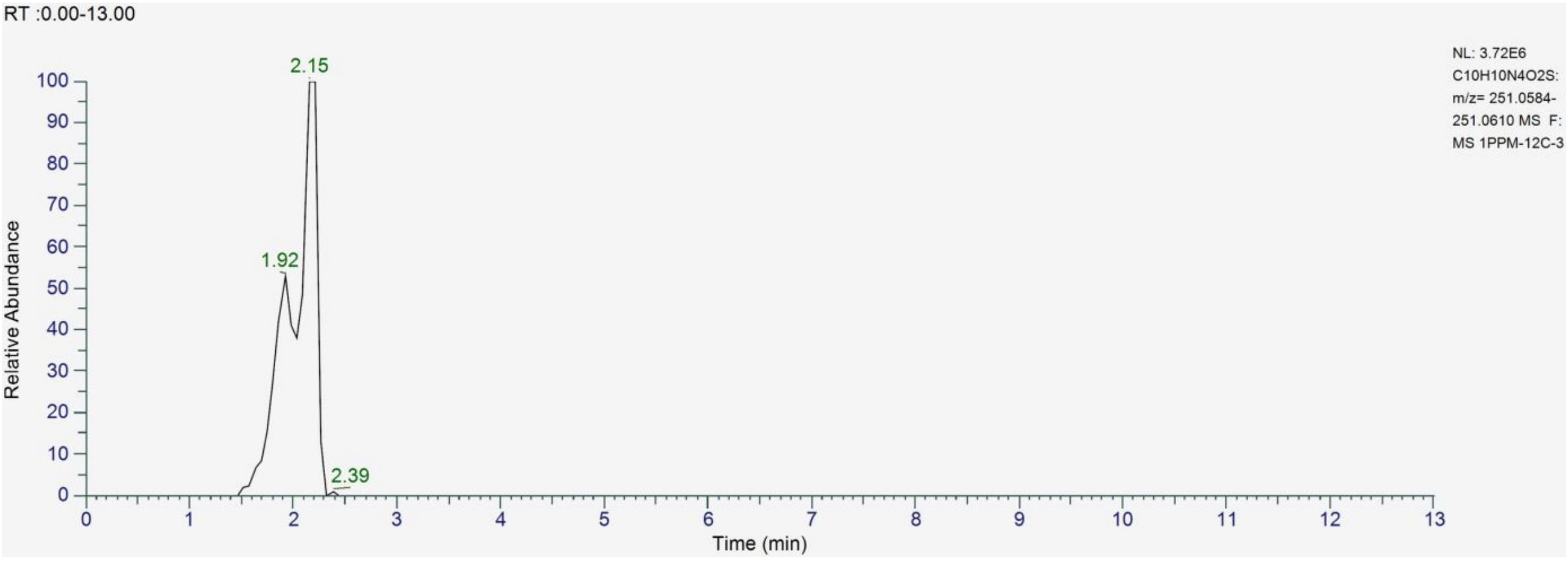

Fig. SI-2. LC-MS analysis of unlabeled sulfadiazine. Extracted ion chromatogram of unlabeled sulfadiazine (SDZ; molecular formula $C_{10}H_{10}N_4O_2S$) acquired using a Thermo Scientific Orbitrap Exploris Series mass spectrometer. The dominant chromatographic peak was observed at RT = 2.15 min, corresponding to the protonated molecular ion $[M + H]^+$ at m/z 251.0584-251.0610, which is consistent with the theoretical exact mass of protonated SDZ $[M + H]^+$, m/z 251.0597.

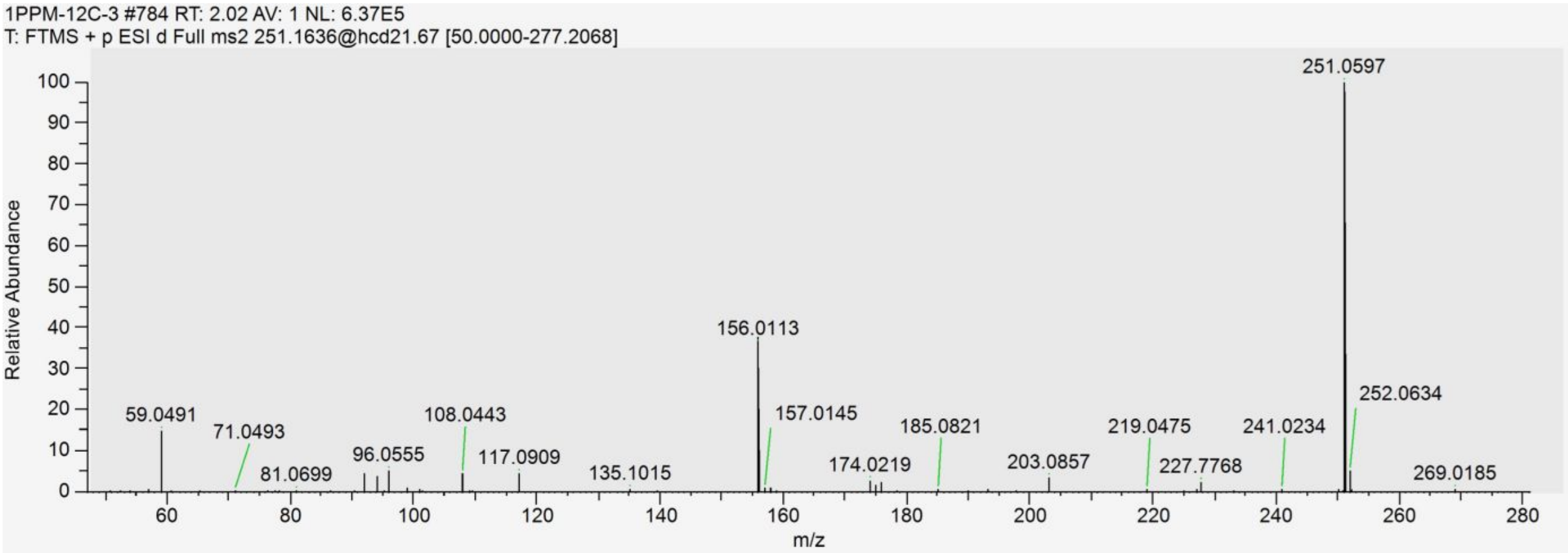

Fig. SI-3. LC-MS spectrum of unlabeled sulfadiazine. Product ion spectrum of unlabeled sulfadiazine (SDZ; $C_{10}H_{10}N_4O_2S$) acquired in positive electrospray ionization mode using a Thermo Scientific Orbitrap Exploris Series mass spectrometer. The protonated molecular ion of SDZ was detected at m/z 251.0597 as the dominant ion, consistent with the theoretical exact mass of $[M + H]^+ = 251.0597$. Diagnostic fragment ions, including m/z 156.0113, 108.0443, and 96.0555, further supported the identity of unlabeled SDZ.

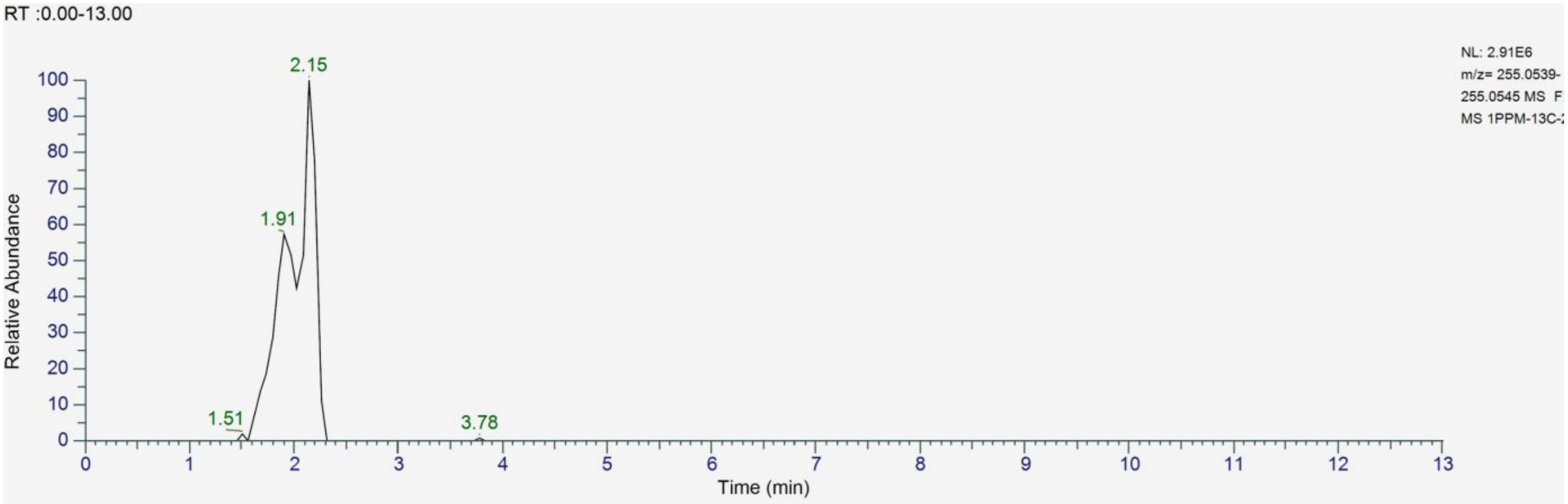

Fig. SI-4. LC-MS analysis of isotope-labeled sulfadiazine. Extracted ion chromatogram of isotope-labeled sulfadiazine ([13C,15N_3]-SDZ; molecular formula 13$C_1$12$C_9$$H_{10}15N_3$14$N_1$$O_2$S) acquired using a Thermo Scientific Orbitrap Exploris Series mass spectrometer. The labeled SDZ showed a dominant chromatographic peak at RT = 2.15 min, corresponding to the expected protonated molecular ion $[M + H]^+$ at m/z 255.0539-255.0545. This value is consistent with the theoretical exact mass of [13C,15N_3]-SDZ $[M + H]^+$ = 255.0542, confirming the expected mass shift caused by incorporation of one 13C atom and three 15N atoms.

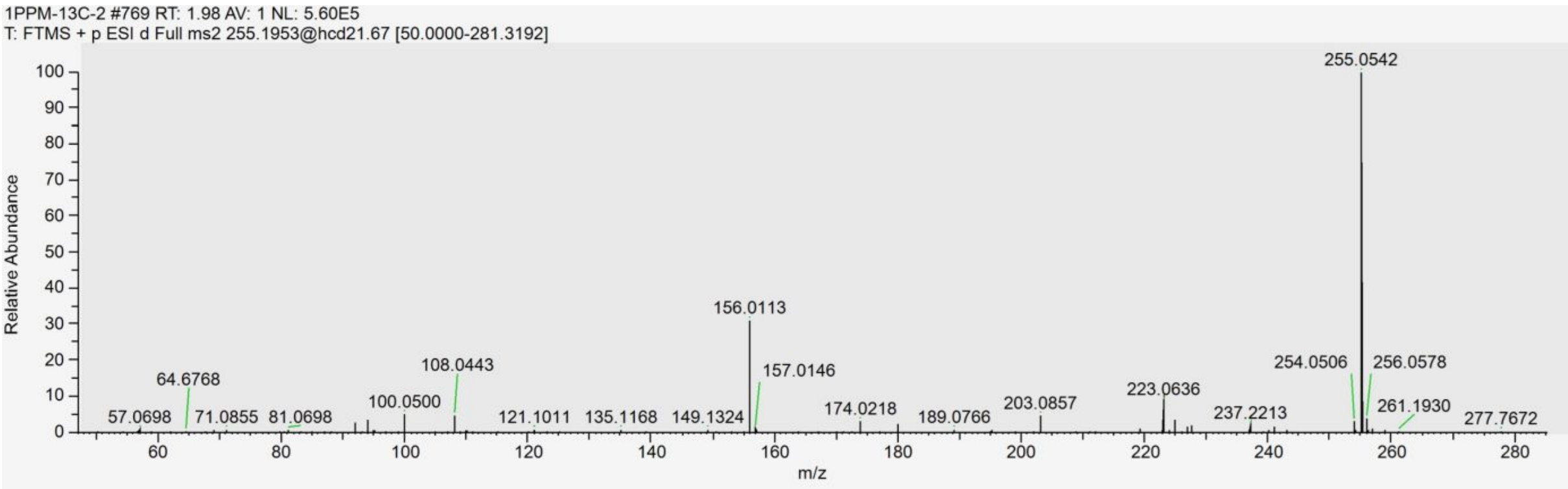

Fig. SI-5. LC-MS verification of isotope labeling position in sulfadiazine. Product ion spectrum of isotope-labeled sulfadiazine ($[^{13}C,^{15}N_3]$-SDZ) acquired in positive ESI mode using a Thermo Scientific Orbitrap Exploris Series mass spectrometer. The precursor ion was detected at m/z 255.0542, consistent with the expected protonated molecular ion of $[^{13}C,^{15}N_3]$-SDZ $[M + H]^+$. Importantly, the diagnostic 2-aminopyrimidine-derived fragment ion was observed at m/z 100.0500, corresponding to the isotope-labeled 2-AP fragment $[^{13}C^{12}C_3H_6{}^{15}N_3]^+$. Compared with the unlabeled 2-AP fragment at approximately m/z 96.0555, this +3.994 Da mass shift is consistent with the incorporation of one ^{13}C atom and three ^{15}N atoms in the 2-AP moiety. The presence of this diagnostic fragment confirms that the ^{13}C and ^{15}N labels were located at the intended position within the 2-aminopyrimidine moiety of SDZ.

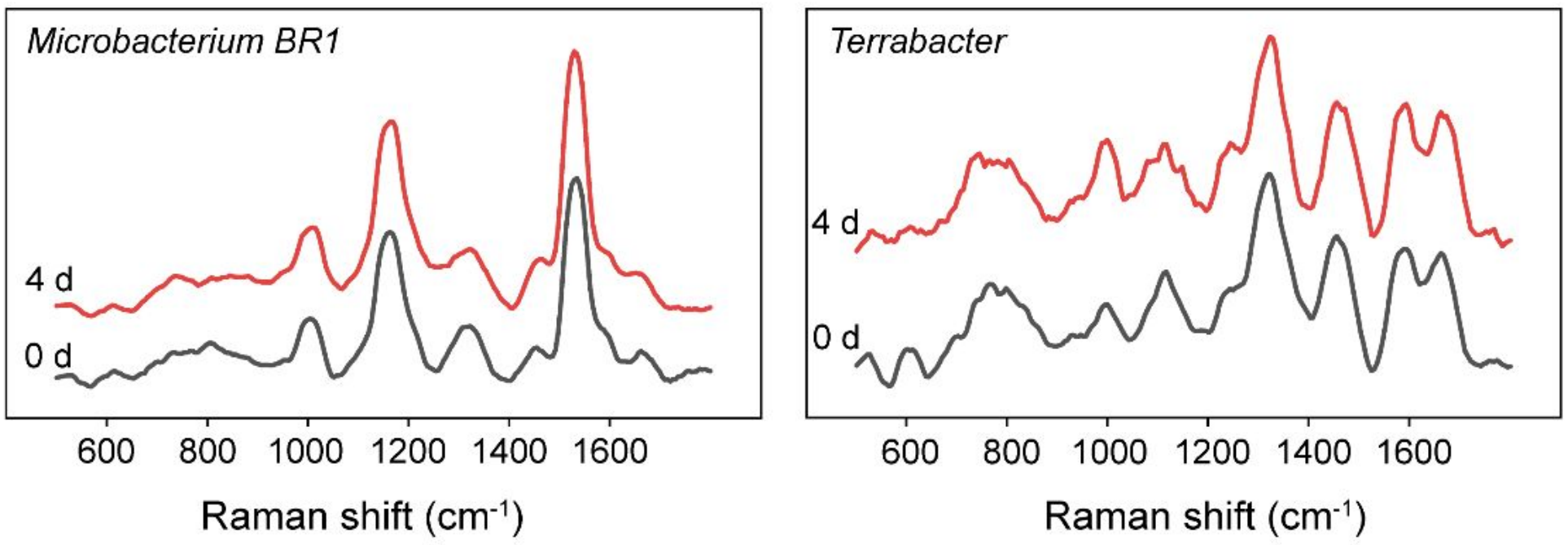

Fig. SI 6. Raman spectra of *Microbacterium* BR1 and *Terrabacter*. Average single-cell Raman spectra of *Microbacterium* BR1 and *Terrabacter* after 4 days of incubation in axenic cultures supplemented with ^{13}C-labeled sulfadiazine. Each spectrum represents the average of 20 single-cell measurements.
